# Systematic Evaluation of Atrous Spatial Pyramid Pooling in U‑Net for Pore Segmentation in Plasma Electrolytic Oxidation Coatings

**DOI:** 10.1021/acs.langmuir.5c01673

**Published:** 2025-06-16

**Authors:** Chi-Wei Chu, Chun-Ming Lu, Wing Kiu Yeung

**Affiliations:** † Department of Materials and Mineral Resources Engineering, 34877 Taipei University of Technology, Taipei 106344, Taiwan; ‡ Institute of Mineral Resources Engineering, 34877 Taipei University of Technology, Taipei 106344, Taiwan

## Abstract

Plasma Electrolytic Oxidation (PEO) coatings enhance the physical and chemical properties of metallic substrates, including corrosion resistance, wear resistance, and thermal stability. These enhancements are strongly influenced by the porous surface morphology of the coatings, which affects the ion transport, stress distribution, and permeability. Accurate quantification of pore structures is essential for understanding interfacial structure–property relationships, yet traditional image segmentation methods often fail to capture the complexity of PEO surfaces in SEM images. This study presents a deep learning-based segmentation framework using U-Net architectures integrated with Atrous Spatial Pyramid Pooling (ASPP) to improve multiscale feature extraction. The performance impact of ASPP placement within different parts of U-Net was systematically evaluated. Results show that modifications to the bridge and decoder paths have the greatest impact on segmentation performance, with a combined modification applying ASPP in both achieving the highest F1 score (0.9360) and the highest IoU (0.8798). Statistical analysis using 5-fold cross-validation, bootstrap confidence intervals, and paired *t*-tests confirmed that only the bridge-modified model (*B*_1×1_) significantly outperformed the baseline (*p* < 0.05). The proposed approach enables high-fidelity pore segmentation and supports advanced microstructural analysis of PEO coatings. By facilitating accurate morphological quantification, it contributes to the understanding of structure–property relationships in interfacial materials and offers a robust tool for future materials characterization workflows.

## Graphical abstract

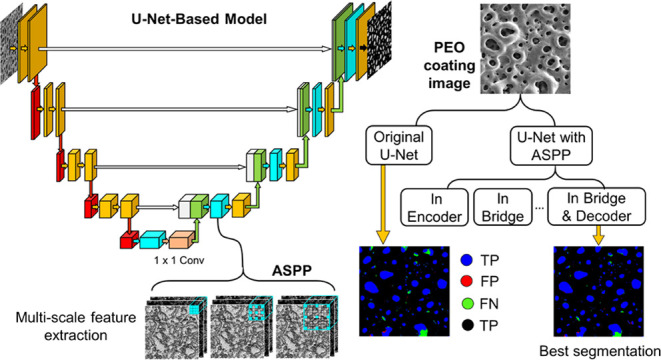

## Introduction

In the era of data-driven innovation, deep learning has significantly expanded the capabilities of computer vision, enabling highly accurate and efficient image analysis.^1^ Among its many applications, semantic segmentation,^2,3^ an advanced technique for pixel-wise classification, has gained prominence for its ability to delineate objects into meaningful categories. This has driven advancements across diverse fields, including those from biomedical imaging^4–6^ to industrial analysis.^7,8^

Plasma Electrolytic Oxidation (PEO) coatings are an emerging class of ceramic-like surface layers that significantly enhance the physical and chemical properties of metallic substrates, including corrosion resistance,^9–11^ wear resistance,^12,13^ thermal stability,^14,15^ and photocatalytic efficiency.^16,17^ These enhancements are largely governed by the porous morphology of the coating, which influences ion or gas transport, local stress distribution, and permeability.^18,19^ Therefore, understanding and quantifying pore structures are essential for correlating the interfacial structure with material performance.

The most thorough approach for analyzing pores in PEO coatings involves micro-CT scanning,^20,21^ which provides high-resolution images of both surface and internal network structures. However, their widespread use is limited by the high cost and accessibility. As a result, SEM image analysis via thresholding remains the most common method.^22^ Yet, due to the complexity of PEO surface morphologies, these traditional methods often require manual corrections, such as adjusting segmentation boundaries or removing artifacts, which are time-consuming, subjective, and prone to human error.^21^

A less-explored method was proposed by Ivasenko et al.,^23^ which used intensity-based segmentation combined with triangle thresholding. However, the model’s performance has not been systematically evaluated. In contrast, recent advances in deep learning, particularly Convolutional Neural Networks (CNNs),^2^ have substantially improved the image segmentation performance across a wide range of domains. Fully Convolutional Networks (FCNs)^24^ were the first to eliminate fully connected layers, enabling pixel-wise prediction with end-to-end learning. SegNet^25^ further advanced this by introducing a symmetrical encoder–decoder structure with pooling index-based upsampling. Among these, the U-Net^26^ and its variants^27^ have demonstrated remarkable success across various domains^5–8,28–31^ due to their encoder–decoder structure and skip connections, which help preserve spatial information even with limited data.^27,31^ U-Net has also been successfully applied to pore segmentation, including bubbles in ice, shale pores, and helium cavities in materials.^7,32,33^ Given the strong performance, U-Net was selected as the base architecture for this study.

However, the wide range of pore sizes and irregular geometries in PEO coatings poses a challenge to standard U-Net, which uses fixed 3 × 3 convolutional kernels. These may be inadequate for capturing both fine details and broader contextual features. To address this, prior research has emphasized the importance of multiscale feature representation in improving segmentation accuracy. For example, Zhou et al.^40^ introduced nested skip pathways in UNet++ to extract hierarchical features, although the design significantly increases the computational load. Gu et al.^41^ (CE-Net) applied multikernel pooling and atrous convolutions in the bridge to enhance contextual information efficiently. Similarly, Mao et al.^42^ (RR-Net) used decomposed convolutions to increase feature diversity, while Liu et al.^43^ (DLGPAFE-Net) incorporated multiscale features within attention mechanisms. PSPNet^44^ further supports the value of multiresolution representation through pyramid-based context aggregation.

Among various strategies, Atrous Spatial Pyramid Pooling (ASPP)^34,35^ has emerged as a lightweight yet effective method. Introduced in the DeepLab series,^34–36^ ASPP applies parallel atrous convolutions with varying dilation rates to capture multiscale context without largely increasing the parameter count. ASPP has since been adapted into U-Net for applications such as medical imaging and remote sensing.^37–39^ For instance, Bansal et al.^45^ applied ASPP from the encoder to the early Decoder, optimizing performance for mobile devices using small dilation rates. Yousef et al.^46^ placed ASPP in the bridge, while Gao and Almekkawy^47^ used dynamic ASPP in the encoder of a nested U-Net to boost performance. Yang et al.^37^ enhanced skip connections using ASPP with channel attention.

While ASPP has demonstrated success in various domains, its optimal integration into U-Net for segmenting the complex and heterogeneous surface features of PEO coatings remains unexplored. This study aims to establish a robust method for SEM image segmentation of PEO coatings while systematically evaluating the impact of integrating ASPP at different stages of the U-Net architecture, encoder, bridge, and decoder for pore segmentation. The modified U-Net variations, termed multiscale atomic convolutional block (MACB) U-Nets, are tested to determine the most effective configuration for improving the segmentation performance.

By improving segmentation fidelity, this study contributes to a more precise evaluation of porosity, pore shape, and spatial distribution, which are essential descriptors in interfacial materials science. The proposed approach supports advanced microstructural analysis and lays the foundation for correlating pore-level features with the macroscopic physical behavior of PEO coatings. This enables future integration of high-throughput image analysis into the material design and property optimization workflows.

## Material and Methods

### Data Acquisition and Preprocessing

The data set used in this study consisted of 200 SEM images of PEO coatings, captured at magnifications ranging from 1000 to 5000× using a JEOL JSM-6510 SEM. The images were acquired from PEO-coated samples prepared under varying processing regimes to capture diverse surface morphologies. To enhance contrast and clarity, brightness adjustments were performed automatically during image acquisition or manually, where necessary.

Each image was cropped to a resolution of 800 × 800 pixels without further resizing to ensure consistency and preserve structural details. The corresponding ground truth segmentation masks were generated via manual annotation by three trained graduate students and authors, and those with prior experience in SEM imaging and PEO coatings conducted annotations using APEER (an online labeling tool) and the GNU Image Manipulation Program (GIMP), which allowed for flexible pixel-level selection and boundary refinement. Annotators followed a standardized labeling protocol, and all annotations were reviewed by a senior researcher to ensure accuracy and consistency across the data set.

Pores were defined as surface-penetrating channels with visible openings including complex structures such as irregularities or protrusions within the openings. Depressed areas without penetration were excluded from the pore category. All annotated masks were binarized, with pore regions assigned a value of 1 and the background assigned 0.

### Basic U-Net Architecture

The U-Net model served as the baseline architecture for this study, as depicted in Figure 1. Figure 1a illustrates the structure of the basic U-Net, which consists of three main components: the encoder, bridge, and decoder. The Basic Convolutional Blocks (BCBs), shown in Figure 1b, form the core of these components. Each BCB consists of two sequential 3 × 3 convolutional layers, each followed by a Rectified Linear Unit (ReLU) activation function,^48^ which is for creating nonlinearity for data fitting. A dropout layer is included after two consecutive convolutional operations to prevent overfitting.^49^

**Figure 1:**
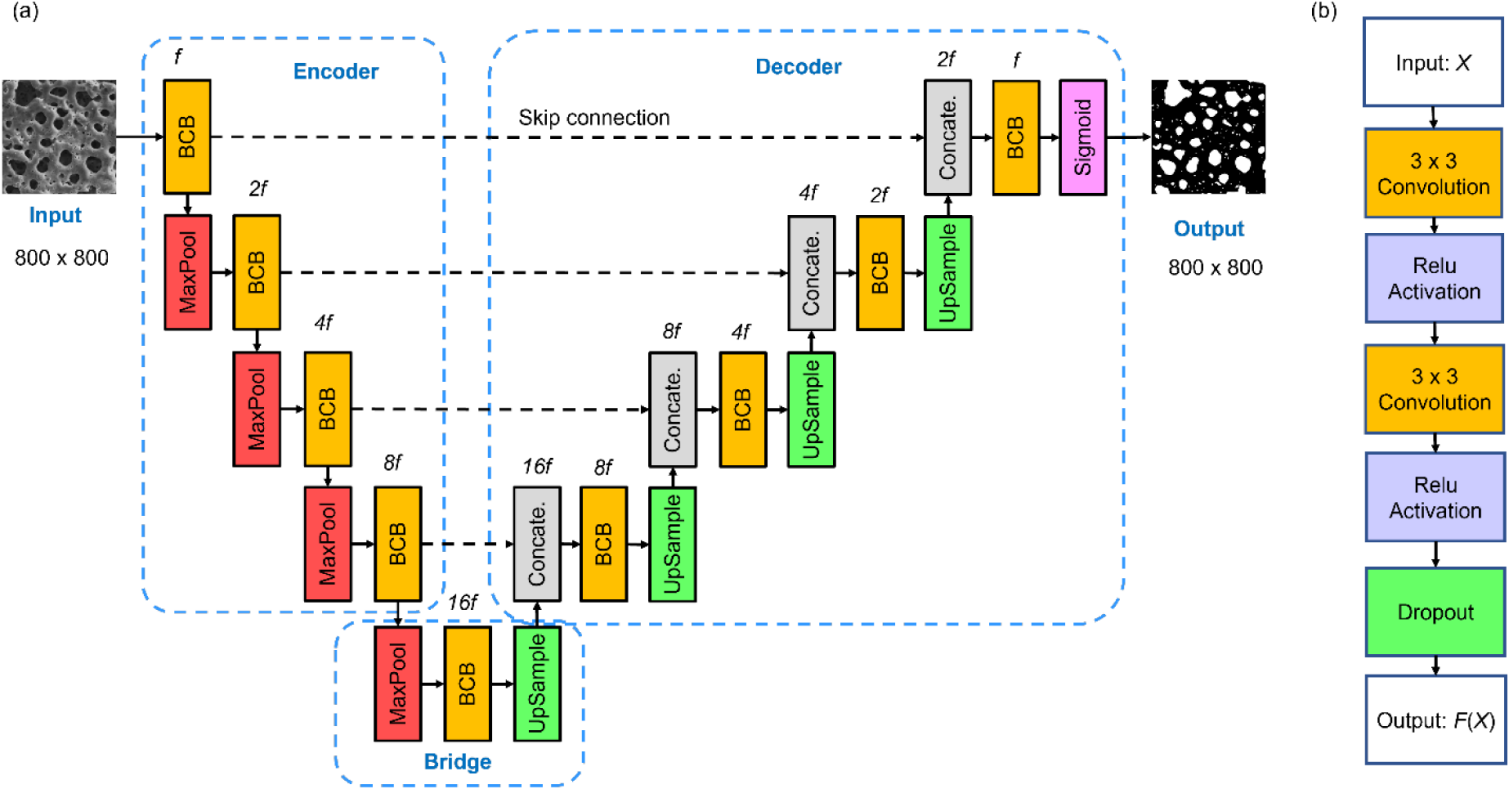
(a) Architecture of the basic U-Net model.^26^ (b) Structure of Basic Convolutional Blocks (BCBs).

The basic U-Net begins with an input layer that processes sliced SEM images of 800 × 800 pixels, normalized to the range [0,1]. The encoder, responsible for extracting multiscale features, consists of a series of convolutional blocks. After each block, a 2 × 2 max-pooling operation with a stride of 2 is applied, reducing spatial dimensions while doubling the number of filters in the subsequent BCB (starting with *f* filters in the first BCB). This down-sampling operation enables the network to capture progressively abstract and high-level features. The bridge acts as an intermediary between the encoder and decoder, processing the downsampled feature maps from the final encoder block before they are up-sampled in the decoder path.

The decoder path mirrors the encoder but focuses on up-sampling the feature maps to restore the original spatial resolution. It begins with a transpose convolution layer, which doubles the spatial size of feature maps while halving the number of filters. The up-sampled feature maps are then concatenated with their corresponding encoder feature maps via skip connections, preserving fine-grained spatial details lost during down-sampling. Each concatenated output is further processed by a BCB. Finally, the output layer applies a 1 × 1 convolution, reducing the depth of the feature maps to a single channel, followed by a sigmoid activation function^48^ to generate the final segmentation mask.

To determine the most suitable baseline model, U-Nets with depths of 7 and 9 BCBs were tested before further modifications.

### Multiscale Atrous Convolutional Block U-Nets

To improve segmentation performance, Atrous Spatial Pyramid Pooling (ASPP) was integrated into the U-Net architecture by replacing standard convolutions in BCB. The ASPP-incorporated structures used in this research are illustrated in Figure 2. Figure 2a illustrates the ASPP convolutional operation, which comprises parallel atrous convolution layers with varying dilation rates. These layers process the input feature maps using atrous filters with different dilation rates, enabling the extraction of both fine-grained local features and broad contextual information. The outputs from these layers are then concatenated and passed through additional convolutions, enabling the network to integrate information across scales. An atrous filter with a dilation rate of *r* enhances the receptive field from k×k to $k_{e}=k+\left(k-1\right)\times \left(r-1\right)$ , where *k* is the original kernel size.

**Figure 2:**
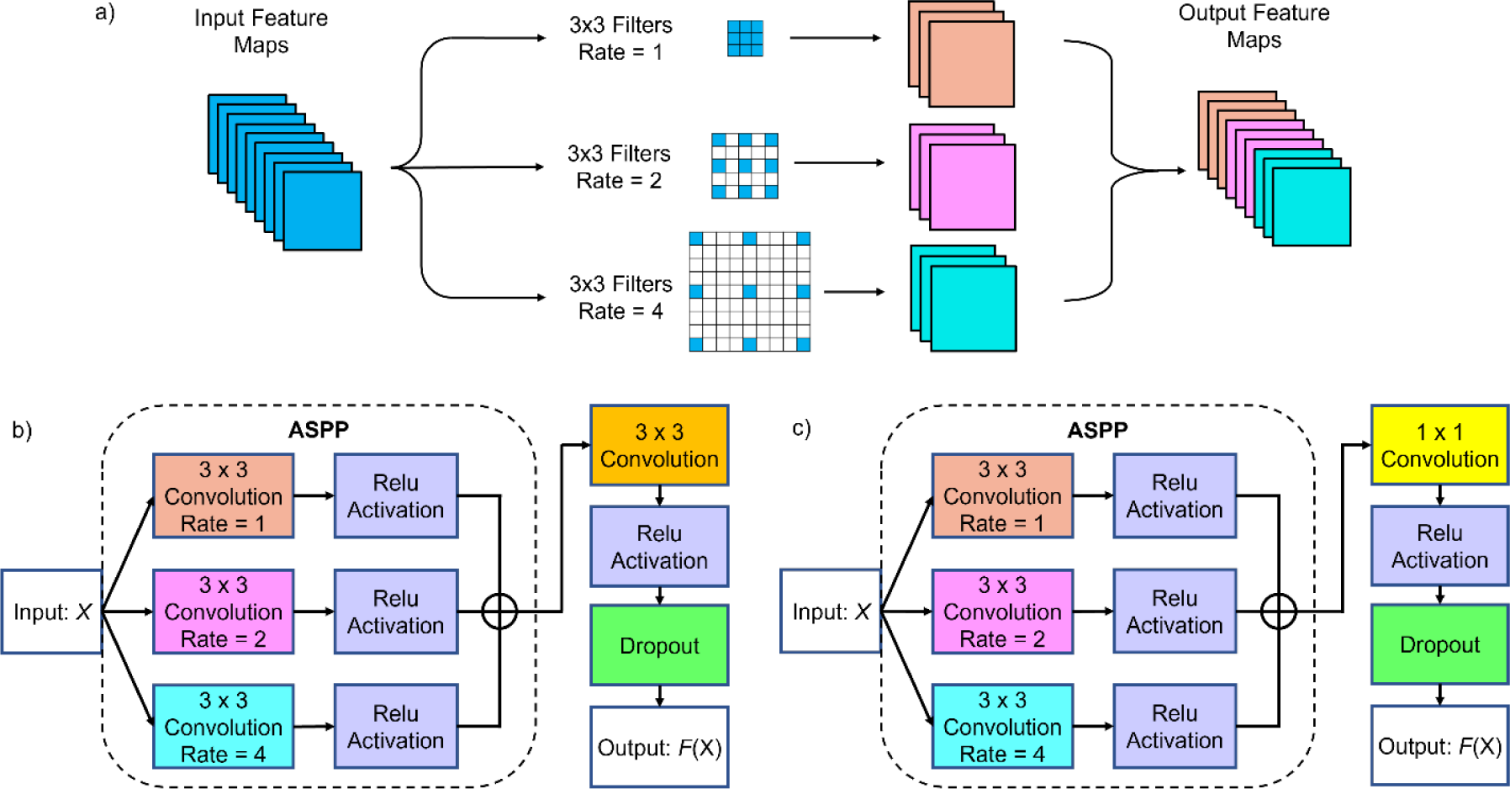
Schematic illustration of multiscale atrous convolutional blocks (MACBs). (a) ASPP convolutional operation, (b) MACB_3×3_, and (c) MACB_1×1_.

The ASPP-revised structure is named multiscale atrous convolutional block (MACB) in this research. Two variations of the MACB were implemented. MACB_3×3_ (Figure 2b) uses three parallel 3×3 atrous convolutions with three different dilation rates, followed by a 3 × 3 convolution to fuse outputs. MACB_1×1_ **(** Figure 2c) uses the same ASPP structure but replaces the final fusion layer with a 1 × 1 convolution, which was the original design in DeepLabv2,^34^ aiming for feature integration and reducing the computational complexity. To manage computational costs, the number of filters in the atrous convolutions was set to one-third of those in standard convolutions (e.g., 8 filters per atrous convolutional branch compared to 24 filters in a standard convolution). The optimal dilation rates were first tuned using a fully MACB_3×3_-modified U-Net, replacing all BCBs in the baseline U-Net 9 architecture before final comparisons. Similar to the BCBs, the filter numbers in the atrous convolutions were doubled after each down-sampling operation and halved after each up-sampling operation.

MACB_3×3_ and MACB_1×1_ were systematically tested in different parts of the U-Net architecture (encoder, bridge, decoder, and their combinations) to determine the optimal placement of the ASPP for segmentation.

### Training Details

Model training was conducted on Google Colab, leveraging an NVIDIA L4 GPU to accelerate deep learning computations. The experiments were implemented by using TensorFlow 2.18.0 and executed in a Python 3.11.11 environment.

Due to the relatively small data set size, 5-fold cross-validation (CV) was employed to ensure robust model evaluation and reduce the risk of overfitting by avoiding reliance on a single test set.^50,51^ In each fold, 40 images were designated as the test set and another 40 as the validation set with the remaining 120 images used for training. These sets were mutually exclusive in each fold. Early stopping was applied to prevent overfitting, and training was terminated if no improvement was observed over 10 consecutive epochs.

To further evaluate the reliability and statistical robustness of our models, we employed two complementary methods: bootstrap resampling and paired *t* tests. For each variant, the 5-fold-level F1 and IoU scores constituted an empirical sampling distribution. We applied nonparametric bootstrap resampling (10,000 iterations) to these values, drawing with replacement to generate distributions of the mean F1 and mean IoU. From each bootstrapped distribution, we derived 95% confidence intervals, defined by the 2.5th and 97.5th percentiles. Concurrently, paired *t* tests were performed on the fold-level metrics to assess the significance of the performance differences between variants.

To enhance model generalization, we augmented the training set with geometric and photometric transformations: rotations (0°, 90°, 180°, and 270°), horizontal and vertical flips, and random brightness/contrast scaling in the range (0.5, 1.5). To evaluate robustness under variable imaging conditions, we also applied brightness/contrast adjustments (same range) to the validation and test sets—while omitting rotations and flips—to emulate real-world variations in SEM acquisition settings.

### Loss Function

Since this study involved binary segmentation, binary cross-entropy was used as the loss function, defined as

1 $$L\left(y,p\right)=-\frac{1}{N}\overset{N}{\underset{i=1}{\sum }}\left[y_{i}\log\left(p_{i}\right)+\left(1-y_{i}\right)\log\left(1-p_{i}\right)\right]$$

where *N* is the number of pixels, *y*_*i*_ is the true class for the *i*-th pixel (0 for background, 1 for pores), and *p*_*i*_ is the predicted probability that the *i*-th pixel belongs to the true class.

The Adam optimizer^52^ was used with a first-moment decay rate (β_1_) of 0.9 and a second-moment decay rate (β_2_) of 0.999. The learning rate was adjusted to enhance training stability, with a clip value of 1 applied to prevent gradient vanishing and explosion.^53^ A minibatch size of 4 was used.

### Quantitative Analysis

Segmentation performance was assessed by classifying predicted pixels into True Positive (TP), False Positive (FP), True Negative (TN), and False Negative (FN). To compare model performances, the F1 score was used as the evaluation metric due to its sensitivity to imbalanced classes. The F1 score is the weighted average of Precision and Recall, providing a balance between precision and recall and offering a comprehensive measurement of segmentation performance:

2 $$\mathrm{Precision}=\frac{\mathrm{TP}}{\mathrm{TP}+\mathrm{FP}}$$

3 $$\mathrm{Recall}=\frac{\mathrm{TP}}{\mathrm{TP}+\mathrm{FN}}$$

4 $$\mathrm{F1}\mathrm{score}=2\times \frac{\mathrm{precision}\times \mathrm{recall}}{\mathrm{precision}+\mathrm{recall}}=\frac{2\mathrm{TP}}{2\mathrm{TP}+\mathrm{FP}+\mathrm{FN}}$$

5 $$\mathrm{DSC}=\mathrm{F1}\mathrm{score}=\frac{2\left(A\cap B\right)}{\left|A\right|+\left|B\right|}=\frac{2\mathrm{TP}}{2\mathrm{TP}+\mathrm{FP}+\mathrm{FN}}$$

where *A* denotes the predicted pore regions and *B* represents the pore regions in the ground truth masks.

Additionally, Intersection over Union (IoU), also known as the Jaccard Index, was used as a supplementary evaluation metric. IoU measures the overlap between predicted and ground truth masks, providing an intuitive measure of segmentation performance:

6 $$\mathrm{IoU}=\frac{A\cap B}{A\cup B}=\frac{\mathrm{TP}}{\mathrm{TP}+\mathrm{FP}+\mathrm{FN}}$$

## Results and Discussion

### Basic U-Nets

Before model comparisons, hyperparameter tuning for basic U-Net models with depths of 7 and 9 BCBs, denoted as U-Net 7 and U-Net 9, respectively, was first conducted using grid search, as detailed in Tables S1 and S2. The 5-fold CV average F1 score was used to assess performance for each combination of filter number, learning rate (LR), and dropout rate. The tested range of hyperparameters included filter numbers for the first convolutional layer from 18 to 40, learning rates from 0.00025 to 0.002, and dropout rates from 0.1 to 0.4. Table S3 summarizes the optimized hyperparameters.

Using these optimized hyperparameters, a final comparison of the test set was conducted, as presented in Table 1.

**Table 1:** Comparison of Segmentation Performance between U-Net 7 and U-Net^a^

Model	Mean F1 Score	95% CI (F1)	*p*-value (F1)	Mean IoU	95% CI (IoU)	*p*-value (IoU)
U-Net 7	0.9195	0.9108–0.9275	-	0.8511	0.8364–0.8649	-
U-Net 9	0.9294	0.9233–0.9356	0.0252*	0.8681	0.8575–0.8789	0.0249*

a Mean F1 scores and Intersection Over Union (iou) were computed from 5-fold cross-validation. 95% Confidence Intervals (CIs) were estimated using 10,000 bootstrap iterations. Paired *t*-tests were used to assess statistical significance; *p* < 0.05 was considered significant and is marked with an asterisk (*).

Table 1 summarizes the segmentation performance of U-Net architectures with different depths. U-Net 9, with increased depth, outperformed U-Net 7, achieving a higher mean F1 score (0.9294 vs 0.9195) and mean IoU (0.8681 vs 0.8511). The 95% confidence intervals for both metrics indicate improved performance and reliability. Importantly, the performance gains were statistically significant, with *p* values of <0.05 for both the F1 score and IoU. Based on these results, U-Net 9 was selected as the baseline architecture for subsequent experiments.

### Multiscale Atrous Convolutional Block U-Nets

Before the optimal placement of ASPP within the U-Net architecture was determined, the dilation rates for the atrous filters were first optimized using the MACB_3×3_ fully modified U-Net, as summarized in Table S4. The optimal dilation rates were determined to be 1, 2, and 4. To evaluate the effect of ASPP in different parts of the U-Net architecture, MACB_3×3_ and MACB_1×1_ U-Nets were compared, as shown in Table 2

**Table 2:** Comparison of Segmentation Performance across U-Net Variants^a^

Model	Mean F1 Score	95% CI (F1)	*p* value (F1)	ΔF1 vs U-Net 9	Mean IoU	95% CI (IoU)	*p*-value (IoU)	ΔIoU vs U-Net 9
U-Net 9	0.9294	0.92328–0.93555	-	-	0.8681	0.85750–0.87893	-	-
E_3×3_	0.9296	0.92186–0.93633	0.9924	+0.02%	0.8684	0.85450–0.88030	0.9898	+0.03%
B_3×3_	0.9323	0.92678–0.93765	0.6425	+0.31%	0.8732	0.86356–0.88263	0.6455	+0.59%
D_3×3_	0.9331	0.92821–0.93778	0.3354	+0.40%	0.8745	0.86606–0.88285	0.3318	+0.74%
EB_3×3_	0.9323	0.92680–0.93703	0.5977	+0.31%	0.8731	0.86329–0.88153	0.5986	+0.58%
BD_3×3_	0.9360	0.93192–0.94004	0.2553	+0.71%	0.8798	0.87256–0.88687	0.2551	+1.35%
ED_3×3_	0.9348	0.92878–0.93993	0.3291	+0.58%	0.8775	0.86711–0.88669	0.3308	+1.08%
EBD_3×3_	0.9339	0.92798–0.93907	0.0364	+0.48%	0.8760	0.86568–0.88515	0.0373	+0.91%
E_1×1_	0.9327	0.92720–0.93755	0.0626	+0.36%	0.8739	0.86433–0.88245	0.0627	+0.67%
B_1×1_	0.9343	0.92893–0.93960	0.0051*	+0.53%	0.8766	0.86731–0.88608	0.0052*	+0.98%
D_1×1_	0.9329	0.92852–0.93642	0.1278	+0.38%	0.8742	0.86661–0.88014	0.1250	+0.70%
EB_1×1_	0.9333	0.92767–0.93850	0.1409	+0.42%	0.8750	0.86515–0.88413	0.1396	+0.79%
BD_1×1_	0.9329	0.92677–0.93900	0.0586	+0.38%	0.8742	0.86357–0.88501	0.0614	+0.70%
ED_1×1_	0.9339	0.92910–0.93809	0.1012	+0.48%	0.8761	0.86762–0.88340	0.1027	+0.92%
EBD_1×1_	0.9326	0.92700–0.93767	0.1298	+0.34%	0.8737	0.86397–0.88267	0.1335	+0.65%
B_1×1__D_3×3_	0.9346	0.92928–0.93694	0.0520	+0.56%	0.8772	0.86840–0.88377	0.0501	+1.05%
E_1×1__B_1×1__D_3×3_	0.9337	0.93158–0.93542	0.2602	+0.46%	0.8755	0.87175–0.87866	0.2603	+0.85%
E_1×1__B_3×3__D_3×3_	0.9339	0.92954–0.93830	0.1160	+0.48%	0.8760	0.86795–0.88136	0.1172	+0.91%

a Mean F1 scores and Intersection Over Union (IOU) were calculated from 5-fold cross-validation. 95% confidence intervals were estimated by bootstrapping (10,000 iterations), and *P*-values were obtained using paired *t*-tests versus the U-Net 9 baseline. An asterisk (*) indicates statistically significant improvement at *p* < 0.05.

Table 2 presents the segmentation performance of U-Net variants modified with MACB_3×3_ and MACB_1×1_ modules. Models were evaluated using 5-fold CV, and the mean F1 score and IoU were computed alongside 95% Confidence Intervals (CIs) estimated via 10,000 bootstrap resampling iterations. The table also reports relative improvements over baseline U-Net 9 and associated *p*-values derived from paired *t*-tests on fold-level scores. To facilitate visual comparison of the statistical spread and overlap between models, an error bar plot depicting the mean F1 scores and IoU with 95% CIs is provided in Figures S1 and S2.

For clarity, models modified with MACB_3×3_ are abbreviated as E_3×3_, B_3×3_, and D_3×3_, corresponding to MACB_3×3_ integration in the encoder, bridge, and decoder paths, respectively. Similarly, the MACB_1×1_ models are labeled as E_1×1_, B_1×1_, and D_1×1_. Combined configurations follow the same naming convention, such as BD_3×3_ for a model with MACB_3×3_ in both the bridge and decoder paths.

The baseline U-Net 9 achieved an F1 score of 0.9294 (95% CI: 0.92328–0.93555) and an IoU of 0.8681 (95% CI: 0.85750–0.87893). All MACB-modified variants demonstrated performance improvements over this baseline, although the degree of improvement and statistical significance varied by the location and type of ASPP module used.

Among the MACB_3×3_ models, decoder-modified variants (D_3×3_, BD_3×3_, ED_3×3_, EBD_3×3_) achieved the most substantial gains (+40–70% F1). Notably, D_3×3_ reached an F1 of 0.9331 (+0.40%, *p* = 0.3354) and an IoU of 0.8745 (+0.74%, *p* = 0.3318), while BD_3×3_ delivered the best overall performance at 0.9360 F1 and 0.8798 IoU (+0.71%, + 1.35%); however, the improvements were not statistically significant (*p* = 0.2553 for F1; *p* = 0.2551 for IoU). In contrast, E_3×3_ showed negligible improvement (+0.02% F1, *p* = 0.9924), suggesting that applying ASPP with 3 × 3 convolution in the encoder path does not provide meaningful benefits for PEO coating segmentation.

This trend suggests that decoder enhancement plays a dominant role in segmentation performance, likely due to its influence in reconstructing detailed spatial features. The bridge-modified B_3×3_ model also performed moderate improvement (+0.31% F1, +0.59% IoU), but again without statistical significance (*p* = 0.6425, *p* = 0.6455), highlighting the bridge path’s importance in aggregating global semantic features even if the gains may vary across folds.

Following MACB_3×3_, MACB_1×1_ modules, similar to the design of DeepLab series,^34–36^ were introduced to explore lightweight alternatives. Despite their reduced complexity, MACB_1×1_ variants still outperformed the baseline. Among them, B_1×1_ achieved the best single-path performance (0.9343 F1, 0.8766 IoU) and was the only model to exhibit statistically significant improvement over U-Net 9 (+0.53%, *p* = 0.0051 for F1; +0.98%, *p* = 0.0052 for IoU), reinforcing the critical role of the bridge path. This is likely due to its central position in the U-Net, where global semantic information can be most effectively aggregated. Additionally, the MACB_1×1_ may serve as a substitute for further down-sampling operations, helping to preserve spatial resolution typically lost during the down- and up-sampling processes.^34^ D_1×1_ also performed well (0.9329 F1, +0.38%, *p* = 0.1278), supporting the importance of decoder-side enhancement. E_1×1_ achieved slightly better performance than E_3×3_ (+0.36% vs +0.02% F1), suggesting that even lightweight 1 × 1 filters can facilitate effective multiscale feature propagation in the encoder without the added complexity or potential early-stage feature fusion introduced by 3 × 3 convolutions.

Interestingly, B_1×1_ outperformed all other MACB_1×1_ variants, even the dridge-based multipath-modified models with additional encoder or decoder modifications. This suggests that oversimplifying the network using multiple 1 × 1 filters across paths may introduce diminishing returns.

These findings motivated the development of hybrid configurations that combine MACB_1×1_ and MACB_3×3_ modules. Three hybrids were evaluated: B_1×1__D_3×3_, combining MACB_1×1_ in the bridge and MACB_3×3_ in the decoder; E_1×1__B_1×1__D_3×3_, adding an encoder MACB_1×1_ to the B_1×1__D_3×3_ structure; and E_1×1__B_3×3__D_3×3_, pairing encoder MACB_1×1_ with the best-performing model, BD_3×3_.

Among them, B_1×1__D_3×3_ (+0.56% F1) outperformed B_1×1_ (+0.53% F1) alone but did not exceed the BD_3×3_ (+0.71% F1) model. The corresponding F1 and IoU improvement was marginally nonsignificant (*p* = 0.0520, *p* = 0.0501), suggesting a trend but not strong enough evidence to confirm a meaningful difference. Both E_1×1__B_1×1__D_3×3_ (+46% F1) and E_1×1__B_3×3__D_3×3_ (+48% F1) showed slightly reduced performance compared to B_1×1__D_3×3_ and BD_3×3_, indicating that adding encoder-side ASPP (E_1×1_) may introduce unnecessary complexity without proportional benefits.

In summary, BD_3×3_ remains the top-performing configuration in terms of raw scores, combining robust contextual representation from the bridge with fine detail recovery from the decoder. However, only B_1×1_ exhibited statistically significant improvements, emphasizing the importance of evaluating both absolute metrics and statistical confidence. This analysis underscores the critical role of ASPP module placement and filter complexity in designing segmentation networks for morphologically diverse structures like PEO pores.

In addition to segmentation performance, the computational cost of the proposed models was evaluated to assess their efficiency, as summarized in Table 3. The training time per epoch was recorded during model development, while the average total training time represents the cumulative duration across all five-folds of cross-validation, incorporating early stopping. The inference time was measured as the average time required to predict a single test image. To facilitate comparison, the relative improvement in F1 score over the baseline U-Net 9 (ΔF1 vs U-Net 9) is also included.

**Table 3:** Computational Cost and F1 Score Improvement (ΔF1) of U-Net Variants with ASPP-Based Modifications^a^

Model	ΔF1 vs U-Net 9	Training time per epoch (s)	Average total training time (min)	Inference time (ms)
U-Net 9	-	96	43.5	25.07
E_3×3_	+0.02%	104	47.5	31.02
B_3×3_	+0.31%	97	66.0	24.49
D_3×3_	+0.40%	135	80.6	46.32
EB_3×3_	+0.31%	104	39.2	31.15
BD_3×3_	+0.71%	135	80.6	46.32
ED_3×3_	+0.58%	142	86.6	52.67
EBD_3×3_	+0.48%	141	55.0	52.67
E _1×1_	+0.36%	97	56.3	31.36
B_1×1_	+0.53%	95	50.7	24.97
D_1×1_	+0.38%	126	56.7	46.49
EB_1×1_	+0.42%	96	56.3	31.20
BD_1×1_	+0.38%	124	40.9	46.59
ED_1×1_	+0.48%	128	69.5	52.22
EBD_1×1_	+0.34%	127	60.5	52.43
B_1×1__D_3×3_	+0.56%	134	47.3	46.58
E_1×1__B_1×1__D_3×3_	+0.46%	134	99.2	52.60
E_1×1__B_3×3__D_3×3_	+0.48%	135	58.5	53.80

a Each model was evaluated by recording the training time per epoch (in seconds), average total training time (in minutes), and inference time (in milliseconds).

Training time per epoch and inference time primarily reflect the computational complexity of each model. Bridge-modified variants such as B_3×3_ (97 s/epoch; 24.49 ms inference) and B_1×1_ (95 s/epoch; 24.97 ms inference) demonstrated comparable or slightly improved efficiency relative to the baseline U-Net 9 (96 s/epoch; 25.07 ms) due to its only one block modification. Encoder modifications introduced moderate overhead (E_3×3_: 104 s; 31.02 ms; E_1×1_: 97 s; 31.36 ms), while decoder-modified models exhibited the most significant increases in both training and inference time (D_3×3_: 135 s; 46.32 ms; D_1×1_: 126 s; 46.49 ms) due to the increased number of filters propagated through skip connections. Multipath models combining encoder, bridge, and decoder enhancements (e.g., E_1×1__B_1×1__D_3×3_: 134 s; 52.60 ms) incurred further increases in complexity.

In contrast, the average total training time reflects both the model complexity and convergence behavior during training. This metric accounts for the number of epochs required to reach optimal performance under early stopping. For example, although B_3×3_ required longer total training time (66.0 min) compared to EB_3×3_ (39.2 min), both achieved the same ΔF1 improvement (+0.31%), suggesting faster convergence for EB_3×3_, likely due to enhanced feature extraction from the added encoder modification. BD_3×3_, the best-performing model in terms of segmentation F1 score (+0.71%), required 80.6 min, indicating a trade-off between the performance gain and training efficiency. Notably, B_1×1_ achieved a substantial improvement (+0.53%) with a relatively low total training and inference time (50.7 min, 24.97 ms), making it a practical and efficient alternative.

In summary, BD_3×3_ remains the optimal choice for maximizing segmentation performance, and B_1×1_ offers a more practical balance between accuracy and efficiency, making it well-suited for real-time applications or resource constrained applications.

### Visual Inspections

Figure 3 presents a visual comparison of segmentation results from the baseline U-Net 9; the encoder-, bridge-, and decoder-modified variants; and the best-performing model (BD_3×3_), based on six representative SEM images. Predicted masks are color-coded as follows: true positives (blue), false positives (red), false negatives (green), and true negatives (black). While all models demonstrate generally acceptable segmentation performance, subtle differences can be observed. The baseline U-Net 9 already produces robust predictions, and the MACB-modified variants exhibit slight improvements in capturing complex pore areas. These observations prompted a more detailed quantitative analysis focused on fine pores (regions with an area below 25 pixels) to objectively assess each model’s ability to detect subtle features.

**Figure 3:**
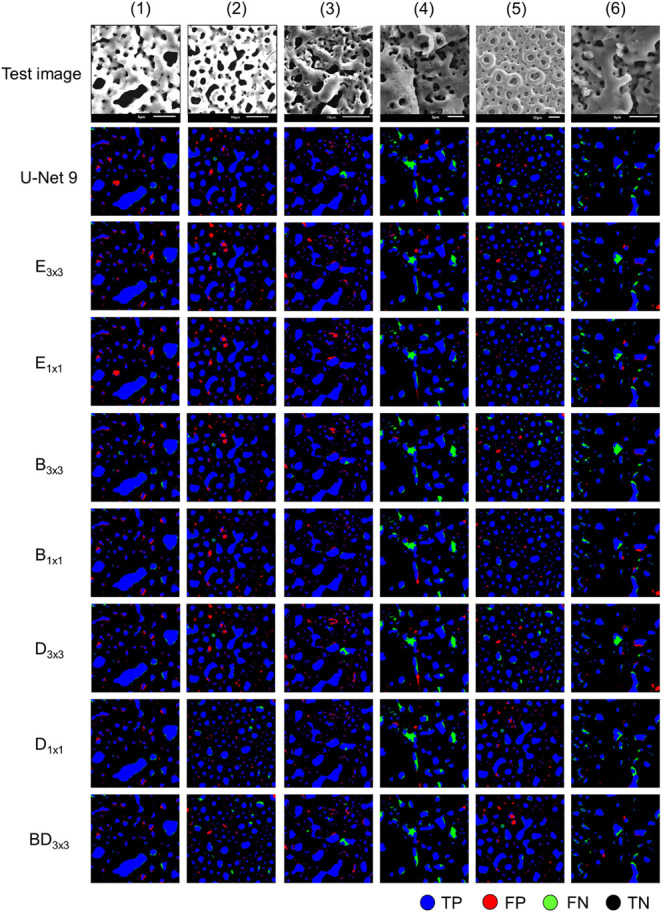
Comparison of U-Net 9, single-path modified U-Net variants, and BD_3×3_ for pore segmentation in PEO Coatings.

As shown in Table 4, all U-Net variants substantially oversegment small pores (area <25 pixels) compared to the ground truth. The ground truth masks contain 1731 small pores, while model predictions range from 3631 (B_1×1_) to 4593 (D_1×1_), more than double the actual count in some cases. Notably, decoder-modified models (D_3×3_ and D_1×1_) exhibit the highest degree of oversegmentation, likely due to their larger skip-connection filters amplifying noise and misinterpreting fine textures as pore boundaries. In contrast, the minimal enhancement in B_1×1_ leads to more conservative segmentation, reducing the misclassification of image noise as pore features. Bridge- and encoder-modified variants tend to produce slightly fewer small pores overall, suggesting that the placement of architectural modifications influences the model’s sensitivity to fine-scale structures.

**Table 4:** Comparison of Small Pore Counts (Area <25 Pixels) Identified in Ground Truth Masks and Predicted by Various U-Net Variants^a^

	Pore count (<25 pixels)
Ground truth	1731
B_1×1_	3631
E_3×3_	3813
E_1×1_	3860
BD_3×3_	3953
B_3×3_	3984
U-Net 9	4053
D_3×3_	4470
D_1×1_	4593

a A total of 40 SEM images were analyzed for each model. The models are listed in the increasing order.

Further comparisons were conducted using masks generated by B_1×1_, BD_3×3_, new students (manual annotation), and the Otsu’s thresholding method, as shown in Figure 4. Two test images were used to illustrate that the segmented masks by B_1×1_ and BD_3×3_ demonstrated markedly superior performance compared to the predictions produced by manual annotation and the Otsu’s method. The manual annotation errors may stem from the new students’ lack of familiarity with the PEO coating structure. The Otsu’s method, relying only on pixel intensity, incorrectly categorized shallow and depressed areas as well as minor dark spots as background, leading to its underperformance.

**Figure 4:**
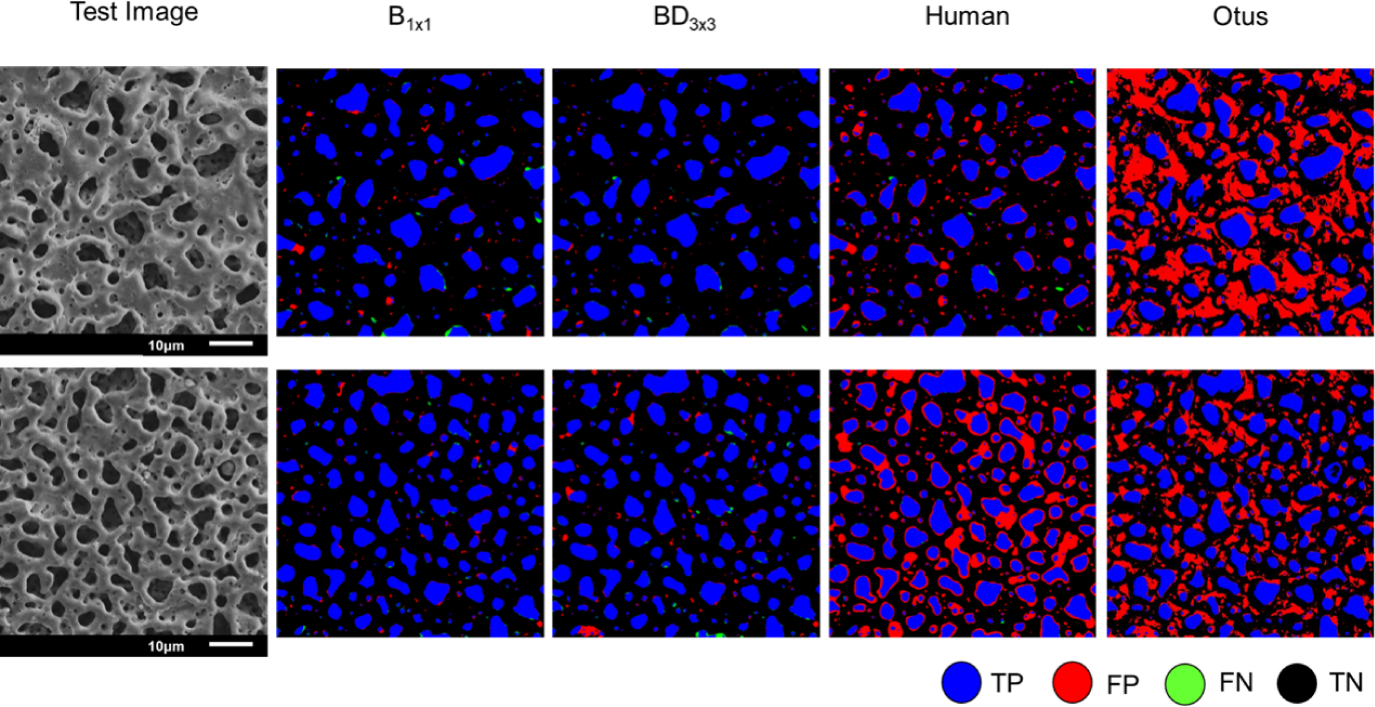
Comparison of B_1×1_, BD_3×3_, human annotation, and Otsu’s method of Otsu for pore segmentation in PEO coatings.

The improved segmentation performance provided by the MACB U-Nets not only enhances the reliability of pore analysis but also contributes to a deeper understanding of the relationship between the pore morphology and the mechanical properties of PEO coatings. This method offers a robust tool for future research and industrial applications, where precise pore analysis is critical.

## Conclusion

This study systematically evaluated the integration of Atrous Spatial Pyramid Pooling (ASPP) into U-Net architectures for the segmentation of pores in PEO coatings. By modifying different parts of the U-Net architecture, namely, the encoder, bridge, and decoder, using multiscale atrous convolutional blocks (MACBs), we identified optimal strategies for enhancing segmentation performance and enabling accurate pore morphology quantification.

Our findings highlight that those modifications to the decoder path, particularly with MACB_3×3_, yield the greatest performance gains, reflecting its critical role in reconstructing fine spatial details. The BD_3×3_ variant achieved the highest F1 improvement (+0.71%) and strong visual performance, while the B_1×1_ variant using pointwise convolution in the bridge was the only model with statistically significant improvements over the baseline, offering a compelling trade-off between accuracy and computational cost.

The small pore analysis revealed that all U-Net variants substantially oversegmented small pores (area <25 pixels), with decoder-modified models (D_1×1_, D_3×3_) showing the highest oversegmentation rates, likely due to the noise amplification caused by integrating ASPP in the decoder path. In contrast, the B_1×1_ model produced more conservative and morphology-consistent results. Additionally, the ASPP-modified U-Net variants outperformed traditional segmentation methods, such as Otsu’s thresholding and human annotations, in terms of both accuracy and reliability.

By enabling high-fidelity segmentation of surface porosity, this work contributes a robust computational tool for the quantitative analysis of interfacial microstructures, supporting efforts to correlate morphology with macroscopic material properties. The framework established here can be extended to other materials with complex surface features, and future research may explore its integration with attention mechanisms or regularization strategies to further enhance generalization and support data-driven materials optimization.

## Supplementary Material



## Data Availability

The full training code for Basic U-Net 9 and the model architectures for all MACB U-Net variants are available at a private GitHub repository. Access will be granted upon reasonable request and the repository will be made public upon publication. GitHub repository: https://github.com/Chi-Wei-Chu/PEO-Coating-Segmentation.

## Notes

Tuning result tables; F1 curves; unpublished works (PDF)

## Abbreviations

BCB: Basic Convolutional Block
MACB: Multiscale Atrous Convolutional Block
E_3×3_: U-Net variant with MACB_3×3_ applied in the encoder
B_3×3_: U-Net variant with MACB_3×3_ applied in the bridge
D_3×3_: U-Net variant with MACB_3×3_ applied in the decoder
E_1×1_: U-Net variant with MACB_1×1_ applied in the encoder
B_1×1_: U-Net variant with MACB_1×1_ applied in the bridge
D_1×1_: U-Net variant with MACB_1×1_ applied in the decoder
EB_3×3_, BD_3×3_, ED_3×3_, EBD_3×3_: U-Net variants with MACB_3×3_ applied in multiple components (e.g., BD_3×3_ indicates MACB_3×3_ in both the bridge and decoder)
EB_1×1_, BD_1×1_, ED_1×1_, EBD_1×1_: U-Net variants with MACB_1×1_ applied in multiple components following the same notation
