# Supplementary material for: Systematic Evaluation of Atrous Spatial Pyramid Pooling in U‑Net for Pore Segmentation in Plasma Electrolytic Oxidation Coatings
Source: Langmuir. 2025 Jun 16;41(25):16368–77. doi: 10.1021/acs.langmuir.5c01673 (PMC12224308; doi:10.1021/acs.langmuir.5c01673)
Supplement: Supplementary file 1 [file la5c01673_si_001.pdf]

Supplementary Materials

■ Systematic Evaluation of Atrous Spatial Pyramid Pooling in U-Net for Pore Segmentation in Plasma Electrolytic Oxidation Coatings

Chi-Wei Chu [b], Chun-Ming Lu [b], Wing Kiu Yeung [a,b*]

[a] Department of Materials and Mineral Resources Engineering, Taipei University of Technology, Taipei 106344, Taiwan
[b] Institute of Mineral Resources Engineering, Taipei University of Technology, Taipei 106344, Taiwan

*Corresponding authors:

E-mail address: vicki.yeung@mail.ntut.edu.tw (Wing Kiu Yeung)

Supplementary Information contains:

Table S1-S4

Figure S1-2

Table S1. 5-Fold cross-validation averaged F1 scores and standard deviations for filter numbers, learning rate, and dropout rate tuning in U-Net 7.

		Dropout 0.1	Dropout 0.2	Dropout 0.3	Dropout 0.4
Filter 18	LR 0.00025	0.9276 ± 0.00493	0.9234 ± 0.00796	0.9128 ± 0.01978	0.8991 ± 0.01743
	LR 0.0005	0.9293 ± 0.00469	0.9244 ± 0.00672	0.9180 ± 0.02374	0.9239 ± 0.00536
	LR 0.001	0.9290 ± 0.00479	0.9292 ± 0.00410	0.9286 ± 0.00529	0.9244 ± 0.00768
Filter 24	LR 0.00025	0.9295 ± 0.00504	0.9258 ± 0.00482	0.9207 ± 0.01011	0.9123 ± 0.02589
	LR 0.0005	0.9290 ± 0.00605	0.9272 ± 0.00820	0.9238 ± 0.01237	0.9190 ± 0.01519
	LR 0.001	0.9288 ± 0.00437	0.9282 ± 0.00258	0.9290 ± 0.00655	0.9225 ± 0.00740
Filter 30	LR 0.00025	0.9267 ± 0.00510	0.9243 ± 0.01347	0.9107 ± 0.01949	0.9117 ± 0.01342
	LR 0.0005	0.9306 ± 0.00482	0.9285 ± 0.00413	0.9262 ± 0.00730	0.9158 ± 0.01485
	LR 0.001	0.9194 ± 0.02169	**0.9311 ± 0.00455**	0.9302 ± 0.00598	0.9299 ± 0.00603
	LR 0.002	0.9048 ± 0.02272	0.9086 ± 0.01885	0.9187 ± 0.01669	0.9088 ± 0.02170
Filter 40	LR 0.00025	0.9258 ± 0.00581	0.9223 ± 0.00889	0.9116 ± 0.00878	0.8993 ± 0.01923
	LR 0.0005	0.9267 ± 0.00667	0.9264 ± 0.00412	0.9263 ± 0.00557	0.9223 ± 0.00284
	LR 0.001	0.9261 ± 0.00613	0.9265 ± 0.00792	0.9253 ± 0.00684	0.9276 ± 0.00624
	LR 0.002	0.8923 ± 0.01966	0.9047 ± 0.02417	0.8860 ± 0.02379	0.8970 ± 0.01948

LR: Learning rate.

Table S2. 5-Fold cross-validation averaged F1 scores and standard deviations for filter numbers, learning rate, and dropout rate tuning in U-Net 9.

		Dropout 0.1	Dropout 0.2	Dropout 0.3	Dropout 0.4
Filter 18	LR 0.00025	0.9333 ± 0.00596	0.9342 ± 0.00608	0.9357 ± 0.00571	0.9307 ± 0.00615
	LR 0.0005	0.9359 ± 0.00607	0.9349 ± 0.00487	0.9365 ± 0.00679	0.9322 ± 0.00475
	LR 0.001	0.9362 ± 0.00608	0.9336 ± 0.00786	0.9273 ± 0.01205	0.9358 ± 0.00578
Filter 24	LR 0.00025	0.9354 ± 0.00592	0.9357 ± 0.00583	0.9368 ± 0.00641	0.9316 ± 0.00867
	LR 0.0005	0.9353 ± 0.00644	**0.9369 ± 0.00423**	0.9355 ± 0.00719	0.9309 ± 0.00826
	LR 0.001	0.9242 ± 0.02404	0.9324 ± 0.00456	0.9271 ± 0.00946	0.9284 ± 0.01218
Filter 30	LR 0.00025	0.9357 ± 0.00671	0.9358 ± 0.00537	0.9346 ± 0.00587	0.9347 ± 0.00652
	LR 0.0005	0.9360 ± 0.00532	0.9352 ± 0.00710	0.9356 ± 0.00488	0.9330 ± 0.00682
	LR 0.001	0.9339 ± 0.00589	0.9242 ± 0.02022	0.9269 ± 0.01045	0.9222 ± 0.00803

LR: Learning rate.

Table S3. Optimized hyperparameters for U-Nets of different depths.

Model	Filter number in the first BCB	Learning rate	Dropout rate	Training time (sec/epoch)
U-Net 7	30	0.001	0.2	95
U-Net 9	24	0.0005	0.2	87

Table S4. 5-Fold cross-validation averaged F1 scores and standard deviations for dilation rates tuning in EBD_{3x3} U-Net.

Dilation rates (1, 2, 3)	Dilation rates (1, 2, 4)	Dilation rates (1, 3, 5)	Dilation rates (1, 4, 6)	Dilation rates (1, 6, 12)
0.9313 ± 0.00574	**0.9323 ± 0.00424**	0.9300 ± 0.00614	0.9316 ± 0.00235	0.9233 ± 0.00507

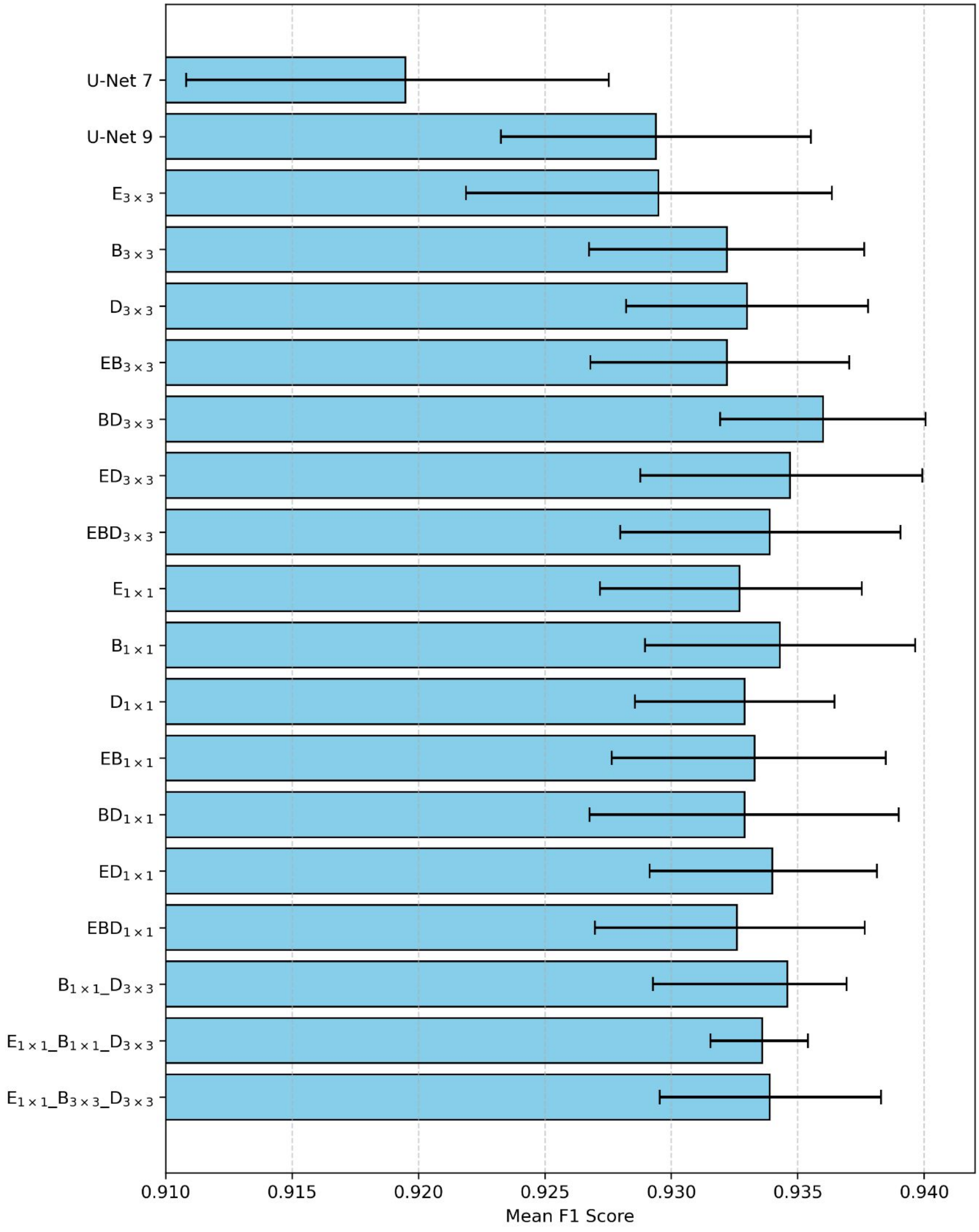

Figure S1. Mean IoU for all U-Net variants with 95% confidence intervals. Error bars were calculated using 10,000 bootstrap resampling iterations.

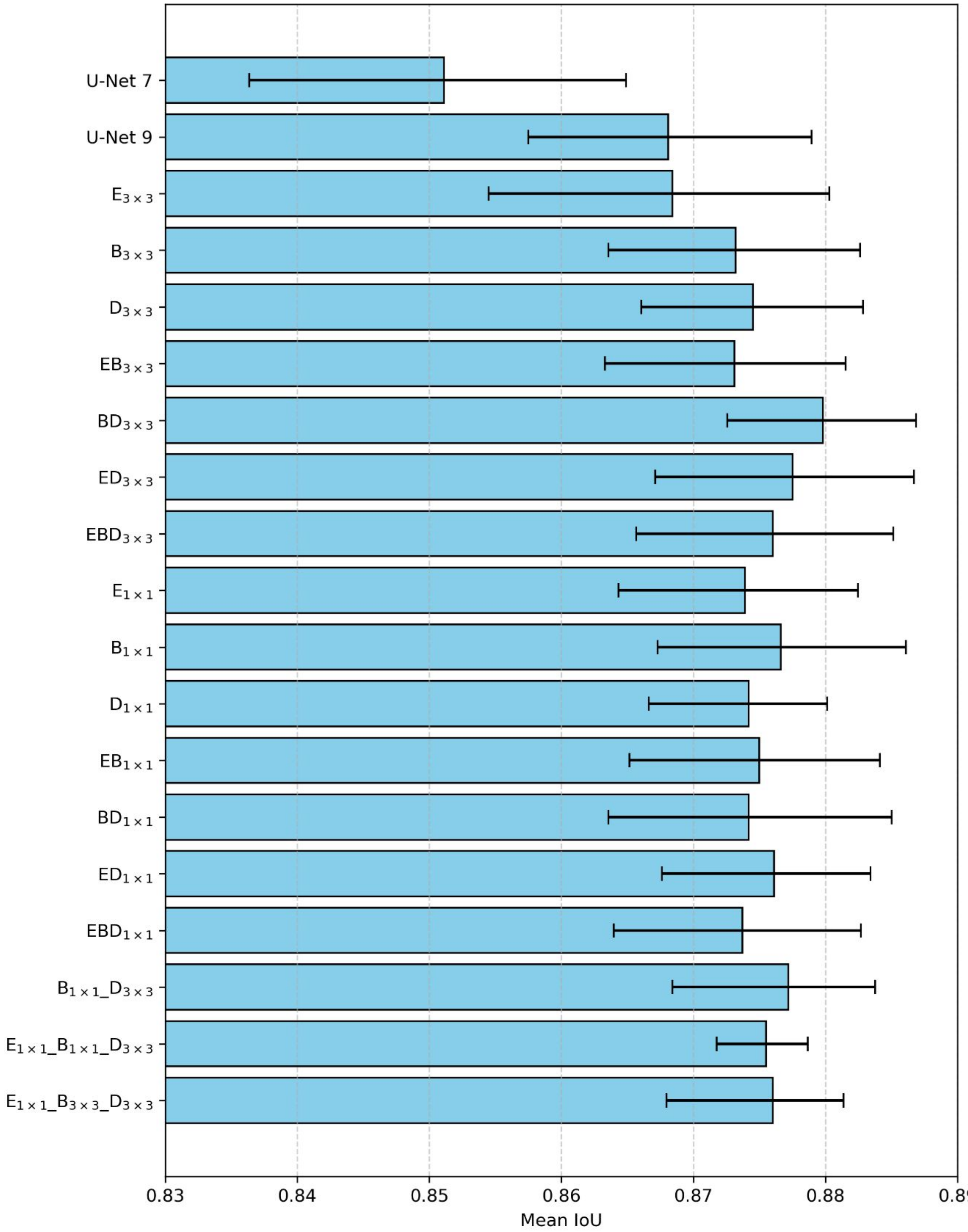

Figure S2. Mean IoU for all U-Net variants with 95% confidence intervals. Error bars were calculated using 10,000 bootstrap resampling iterations.
